# Role of stretch-activated channels in light-generated action potentials mediated by an intramembrane molecular photoswitch

**DOI:** 10.1186/s12967-024-05902-4

**Published:** 2024-11-27

**Authors:** Chiara Florindi, Vito Vurro, Paola Moretti, Chiara Bertarelli, Antonio Zaza, Guglielmo Lanzani, Francesco Lodola

**Affiliations:** 1 Department of Biotechnology and Biosciences, University of Milano-Bicocca, P.za della Scienza 2, 20126 Milan, Italy; 2 Center for Nano Science and Technology, Istituto Italiano di Tecnologia, Milan, Italy; 3 Department of Chemistry, Materials and Chemical Engineering “Giulio Natta”, Politecnico di Milano, Milan, Italy; 4 Department of Physics, Politecnico di Milano, Milan, Italy

**Keywords:** Light stimulation, Molecular photoswitches, Cardiac cell modulation, Stretch-activated channels

## Abstract

**Background:**

The use of light to control the activity of living cells is a promising approach in cardiac research due to its unparalleled spatio-temporal selectivity and minimal invasiveness. Ziapin2, a newly synthesized azobenzene compound, has recently been reported as an efficient tool for light-driven modulation of excitation-contraction coupling (ECC) in human-induced pluripotent stem cells–derived cardiomyocytes. However, the exact biophysical mechanism of this process remains incompletely understood.

**Methods:**

To address this, we performed a detailed electrophysiological characterization in a more mature cardiac model, specifically adult mouse ventricular myocytes (AMVMs).

**Results:**

Our in vitro results demonstrate that Ziapin2 can photomodulate cardiac ECC in mature AMVMs without affecting the main transporters and receptors located within the sarcolemma. We established a connection between Ziapin2-induced membrane thickness modulation and light-generated action potentials by showcasing the pivotal role of stretch-activated channels (SACs). Notably, our experimental findings, through pharmacological blockade, suggest that non-selective SACs might serve as the biological culprit responsible for the effect.

**Conclusions:**

Taken together, these findings elucidate the intricacies of Ziapin2-mediated photostimulation mechanism and open new perspectives for its application in cardiac research.

**Supplementary Information:**

The online version contains supplementary material available at 10.1186/s12967-024-05902-4.

## Background

The use of light to manipulate cellular behavior marks a transformative breakthrough in cardiac research, providing unparalleled advantages, including precise spatial and temporal control with minimal disruption to cellular function [1].

Optogenetics has revolutionized the field, allowing the investigation of fundamental mechanisms of cardiac electrophysiology and offering crucial insights into arrhythmia mechanisms and potential therapies [1–13].

**Scheme 1:**
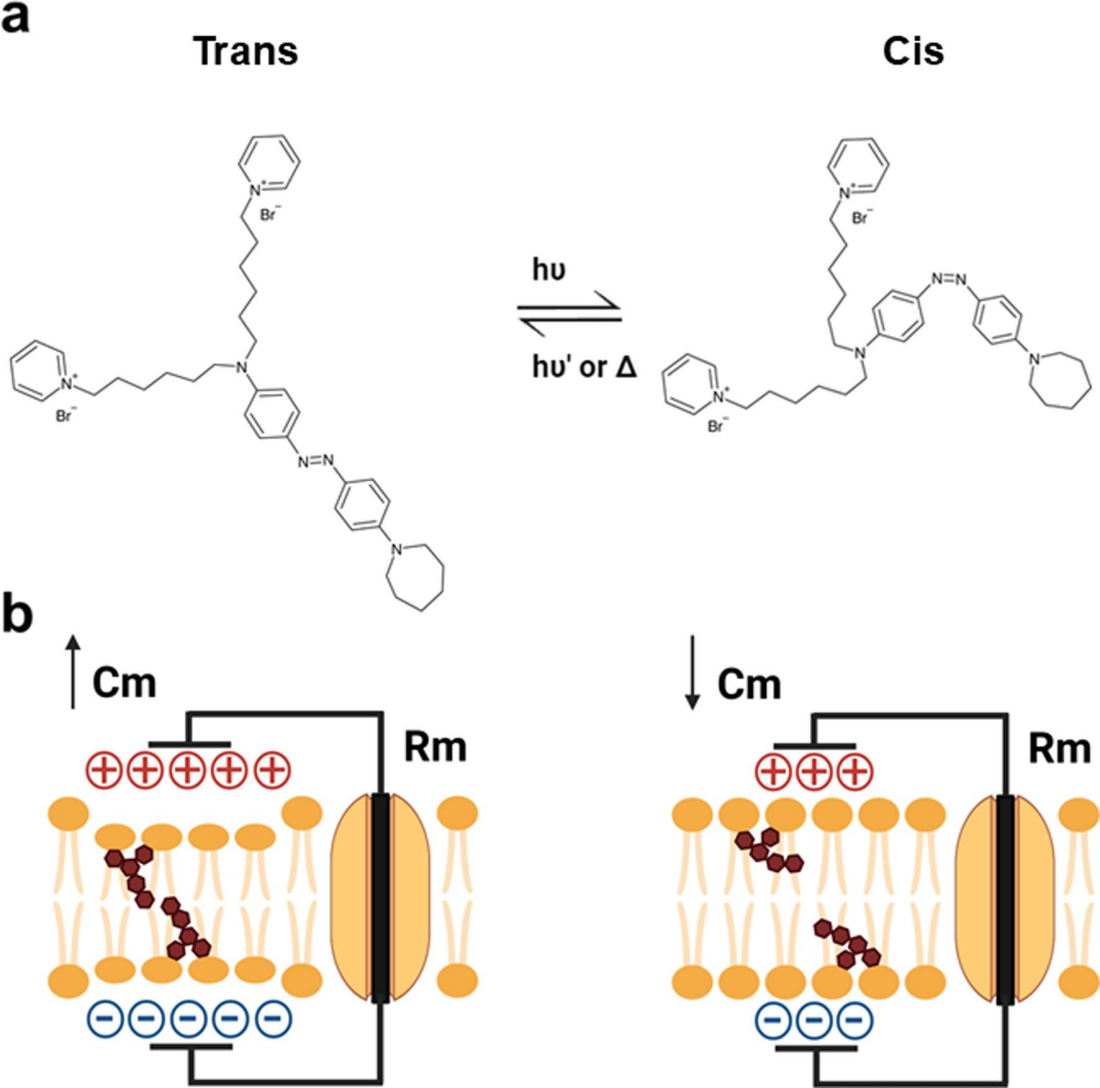
Cartoon depicting the Ziapin2 trans-to-cis photostimulation process. (**a**) Ziapin2 molecular structure and its light-induced isomerization. (**b**) In the trans conformation, Ziapin2 molecules anchor to the opposite leaflets of the membrane, forming dimers. This causes a local thinning of the membrane, resulting in an increase in membrane capacitance (left). Upon millisecond pulses of visible light (λ = 470 nm), Ziapin2 undergoes isomerization into its cis form. In this configuration, the hydrophobic tails of opposing molecules are too far apart to dimerize, causing a rapid drop in capacitance due to membrane relaxation (right)

An alternative strategy relies on light-sensitive transducers based on organic small molecules. In this context, our research team developed Ziapin2, a novel photoswitch featuring an aminoazobenzene chromophore and an amphiphilic structure, whose efficacy has been proven both in vitro and ex vivo in several experimental models [14–20]. Ziapin2 can partition into cell membranes and change its conformation when exposed to visible light (470 nm). This photoisomerization perturbs the membrane bilayer structure, affecting cell membrane thickness and causing a change in membrane capacitance that modulates membrane potential (Scheme 1).

Notably, when added to human-induced pluripotent stem cells–derived cardiomyocytes (hiPSC-CMs) cultures, Ziapin2 enabled photoactivated action potential triggering via molecule isomerization [19]. The electrical activity correlates with changes in Ca^2+^ dynamics and an increase in the contraction rate, suggesting the possibility to control the whole excitation-contraction coupling (ECC) process. The photopacing efficacy of this approach has been further extended to a cardiac microphysiological model that mimics the cellular organization and substrate mechanical properties of native cardiac tissue, proving that Ziapin2 could be a viable tool for the modulation of ECC with precise spatial and temporal control [20].

These encouraging findings imply that light-sensitive molecules hold potential for non-genetic, contactless, and temperature-independent regulation of cardiac electrical activity, indicating their strong suitability for pacing and anti-arrhythmic purposes. However, despite its promising applications, the underlying biophysical mechanisms driving this light-induced modulation remain poorly understood, presenting a critical frontier in optimizing this transformative approach.

In the present work, we performed an in-depth electrophysiological analysis of the light-induced modulation mediated by Ziapin2 in adult mouse ventricular myocytes (AMVMs). AMVMs provide a model that reflects mature and physiologically relevant characteristics of adult human cardiac tissue [21]. Within this context, we characterized the molecular mechanism of action, evaluating its potential interactions with key elements of the ECC system, such as membrane receptors and transporters. Finally, we focused on the biophysical processes that trigger action potential generation, studying the involvement of channels whose open probability is strictly dependent on Ziapin2-mediated membrane perturbation.

## Methods

### Cardiomyocyte isolation

Mice were euthanized by cervical dislocation under anaesthesia with ketamine-xylazine (130–7.5 mg/kg i.p) and ventricular cardiomyocytes were isolated by using a manual perfusion method [22, 23].

Briefly, while the heart was still in situ, the right ventricle was injected with 7 mL of EDTA buffer solution (130 mM NaCl, 5 mM KCl, 0.5 mM NaH_2_PO_4_, 10 mM HEPES, 10 mM Glucose, 10 mM 2,3-Butanedione Monossime, BDM, 10 mM Taurine, 5 mM EDTA; pH = 7.8 with NaOH); the ascending aorta was then clamped and the heart was transferred to a dish containing EDTA buffer solution.

Digestion was achieved by sequential injections into the left ventricle of 10 mL EDTA buffer solution, 6 mL of perfusion buffer solution (130 mM NaCl, 5 mM KCl, 0.5 mM NaH_2_PO_4_, 10 mM HEPES, 10 mM Glucose, 10 mM 2,3-Butanedione Monossime, BDM, 10 mM Taurine, 1 mM MgCl_2_; pH = 7.8 with NaOH) and 20 to 30 mL of Liberase buffer solution (0.1 mg/ml Liberase in Perfusion Buffer + 0.02 mg/ml Trypsin EDTA and 6.25 µM CaCl_2_).

Cellular dissociation was completed by gentle trituration, while enzyme activity was quenched by adding 10 mL of stop buffer solution (perfusion Buffer + 10% FBS). The resulting suspension was filtered, centrifuged and resuspended in calcium free solution (130 mM NaCl, 5.4 mM KCl, 0.4 mM NaH_2_PO_4_, 0.5 mM MgCl_2_, 25 mM HEPES, 22 mM Glucose; pH = 7.4 with NaOH). Finally, the physiological extracellular Ca^2+^ concentration was gradually reestablished by adding to the cell suspension small volumes at increasing CaCl_2_ concentration.

### Ziapin2 synthesis and uptake process

Ziapin2 was synthesized as described previously [15–20]. Micromolar concentrations (25 µM) of the molecule were added to AMVMs previously seeded in a petri dish mounted a Nikon Eclipse TE200 inverted microscope. The cells were incubated with Ziapin2 at room temperature for 7 min, avoiding direct exposure to light. Following incubation, the cells were perfused with fresh extracellular solution to remove any uninternalized molecule.

### Electrophysiological recordings

Only quiescent, Ca^2+^-tolerant, rod-shaped AMVMs with clear cross striations were used for electrophysiological recordings. Both voltage and current-clamp experiments were performed by the ruptured-patch version of the whole-cell mode [24].

Cells were perfused (at 36 °C) with an extracellular solution containing (in mM): 154 NaCl, 4 KCl, 5 HEPES NaOH, 1.2 or 2 mM CaCl_2_, 1 mM MgCl_2_, 5.5 glucose; pH = 7.35 with NaOH. Patch pipettes (tip resistance of 3.5–4.5 MΩ) were filled with an intracellular solution containing (in mM): 110 K-aspartate, 23 KCl, 3 MgCl_2_, 0.04 CaCl_2_, 0.1 EGTA KOH (10^− 7^ Ca^2+^-free), 5 Hepes KOH, 0.4 Na^+^-GTP, 5 Na^+^-ATP, 5 Na^+^-phosphocreatine; pH = 7.3 with KOH. Correction for the liquid junction potential between pipette and bath solution was performed.

Electrically induced action potentials were elicited by intracellular current injection through patch electrodes using depolarizing pulses.

The sodium-calcium exchange current (I_NCX_) was elicited by the caffeine (10 mM) pulse applied after a loading train of V steps (− 40 to 0 mV at 1 Hz) and recorded as a transient inward current. To quantify the decay phase, the curve of I_NCX_ was fitted with a mono-exponential function. The area under the I_NCX_ current curve was integrated to estimate the sarcoplasmic reticulum (SR) Ca^2+^ content (CaSR) [23]. To avoid Ca^2+^ influx during the SR emptying, caffeine was dissolved in Ca^2+^-free solution (containing 1 mM EGTA CsOH).

The recorded signals were amplified by MultiClamp 700A (Molecular Devices, Sunnyvale, CA, United States), digitized at 20 kHz (Axon Digidata 1440 A, Molecular Devices), and filtered at 10 kHz.

### Capacitance recordings

Capacitance measurements were performed as previously described [19]. Briefly, a double sinusoidal voltage clamp signal was applied to the cell in whole-cell configuration. The response current signal was acquired, and membrane capacitance and resistance were extracted fitting the current with a custom code implemented in MATLAB (MathWorks). The capacitance value was extracted in dark condition and during light stimulation. A 250 ms pulse was used to consider mainly the effect related to Ziapin2 photoisomerization.

### Acquisition and analysis of AMVMs contractile behavior

A Nikon Eclipse Ti inverted microscope was used to stimulate (Lumencor Spectra X, λ_ex_ = 470 nm, 20X objective, Pobj = 30 mW/mm^2^) and acquire video of the AMVMs contraction frequency. Contractile behavior was analyzed using a custom-built algorithm, implemented in MATLAB (MathWorks) [19]. The approach is based on the contraction-induced retraction of cell body towards nucleus and is effective even if there is no displacement of cell extremities or if the cellular edge cannot be detected properly.

The user defines one or more regions of interest (ROI) using bounding boxes. Each bounding box should contain one single cell. For each ROI a set of features is identified and tracked across video frames by means of Kanade-Lucas-Tomasi algorithm [25, 26]. Since the ROI is delimiting a cell, the tracked features are expected to belong to the cell body. Averaging the estimated motion fields of all these feature points returns a mean geometric transformation, which can be applied to the bounding box delimiting the ROI. The area of this bounding box is measured over time and presents minima at cellular contractions. The number of minima per time interval yields an estimate of the cell contraction rate. Data were analyzed with Origin 9.0 (OriginLab Corporation).

### Photostimulation protocol

Illumination of cells during electrophysiological experiments was provided by a collimated Light-emitting diode (LED, Thorlabs) coupled to the fluorescence port of a Nikon Eclipse TE200 inverted microscope. The external light source was characterized by a maximum emission wavelength of 470 nm to match the molecule absorption spectrum. The illuminated spot on the sample has an area of 0.27 mm [2] and a photoexcitation density of 50 mW/mm^2^, as measured with an optometer at the output of the microscope objective.

### Statistical analysis

GraphPad Prism 8 (GraphPad software, San Diego, CA, USA) was used for statistical analysis.

Normality of distribution was assessed using D’Agostino-Pearson’s normality test. To compare two sample means, either the student’s t-test and the Mann-Whitney U-test were used for continuous or categorical data, respectively. To compare more than two sample means, one-way ANOVA (with Tukey correction) or Kruskal-Wallis (with Dunn’s correction) were used for continuous or categorical data, respectively.

In figures, whenever feasible, individual data points were plotted, to illustrate dispersion, along with the sample mean ± SEM. Whenever the threshold for statistical significance (*p* < 0.05) was achieved, the actual p-value for the comparison was reported as an index of robustness. For each experiment, the number of preparations or cells (n) and the number of animals from which they were obtained (N) are indicated in the respective figure legend.

## Results and discussion

### Ziapin2 modulates excitation-contraction coupling in mouse AMVMs

First, we tested Ziapin2 efficacy in our experimental model. To this aim, AMVMs were loaded with 25µM Ziapin2 and whole-cell patch clamp measurements in current clamp mode (I = 0) were performed.

In these experiments, the cells were stimulated with either short (20 ms) or long (200 ms) light pulses at a power density of 50 mW/mm^2^. As expected, the molecule’s photoisomerization induced a biphasic modulation of the membrane potential (Vm) with a rapid hyperpolarization peaking approximately 10 ms after the light onset, followed by a delayed depolarization (teal traces in Fig. S1). No Vm alterations were detected in vehicle-treated AMVMs subjected to the same photostimulation protocols (grey traces in Fig. S1).

Previous in vitro observations and in silico predictions explained the Vm variation as a consequence of changes in membrane capacitance caused by Ziapin2-mediated membrane thickness modulation (Scheme 1) [15–19].

**Fig. 1:**
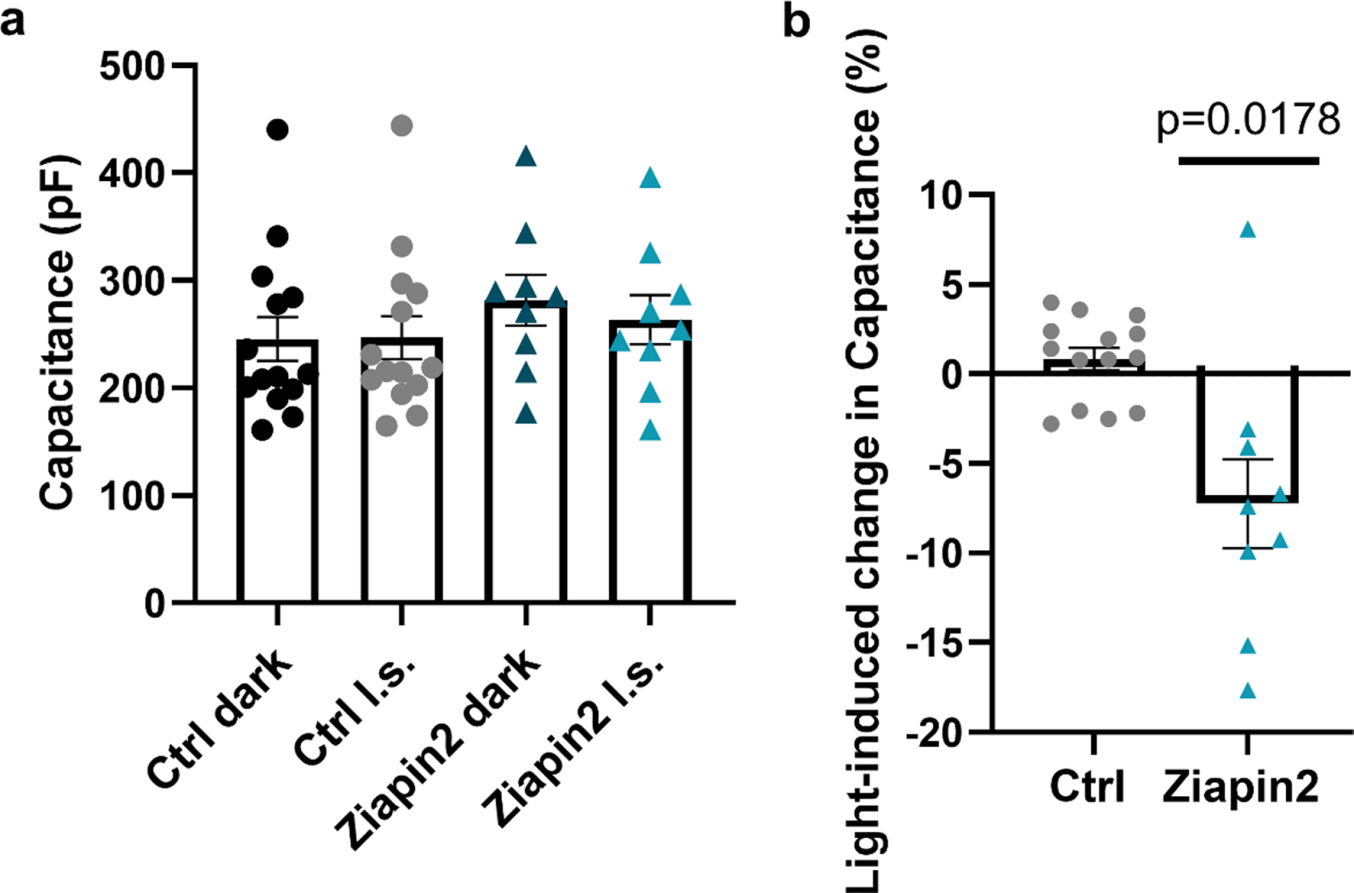
Ziapin2-mediated modulation of membrane capacitance in AMVMs. **(a)** Evaluation of capacitance changes in vehicle- (Ctrl) or 25 µM Ziapin2-loaded AMVMs in dark and upon photostimulation at 50 mW/mm^2^ light power density. Ctrl dark: 245.7 ± 20.5 pF, *n* = 14; Ctrl l.s: 246.9 ± 19.9 pF, *n* = 14; Ziapin2 dark: 281.7 ± 23.4 pF, *n* = 9; Ziapin2 l.s: 263.6 ± 23 pF, *n* = 9. **(b)** Percentage of variation in capacitance upon light stimulation (Δ% Ctrl: 0.84 ± 0.6, *n* = 14; Δ% Ziapin2: -7.2 ± 2.4, *n* = 9). Data are represented as mean ± SEM. Ctrl: *N* = 6; Ziapin2: *N* = 4. Statistical comparison in panel b was performed using the paired Student’s t-test (Ziapin2) or the Mann-Whitney test (Ctrl), depending on the normality of the data distribution

This was confirmed in AMVMs through capacitance measurements performed both in dark and under visible light illumination (Fig. S2). In dark, Ziapin2-loaded AMVMs exhibit a significant increase in capacitance (+ 14.6%, from 245.7 ± 20.5 pF to 281.7 ± 23.5 pF) assigned to the bilayer thinning caused by Ziapin2 trans-dimerization (Fig. 1a). Upon light stimulation, a partial return toward steady-state capacitance values (-6.8%, mean decrease ± SEM: -18.10 ± 6 pF) was observed, due to light-induced trans→cis photoconversion and consequent membrane relaxation towards its native thickness (Fig. 1b). Consistent with Vm modulation data (Fig. S1), no light-dependent effects on capacitance were noticed in vehicle-treated cells (Fig. 1a).

**Fig. 2:**
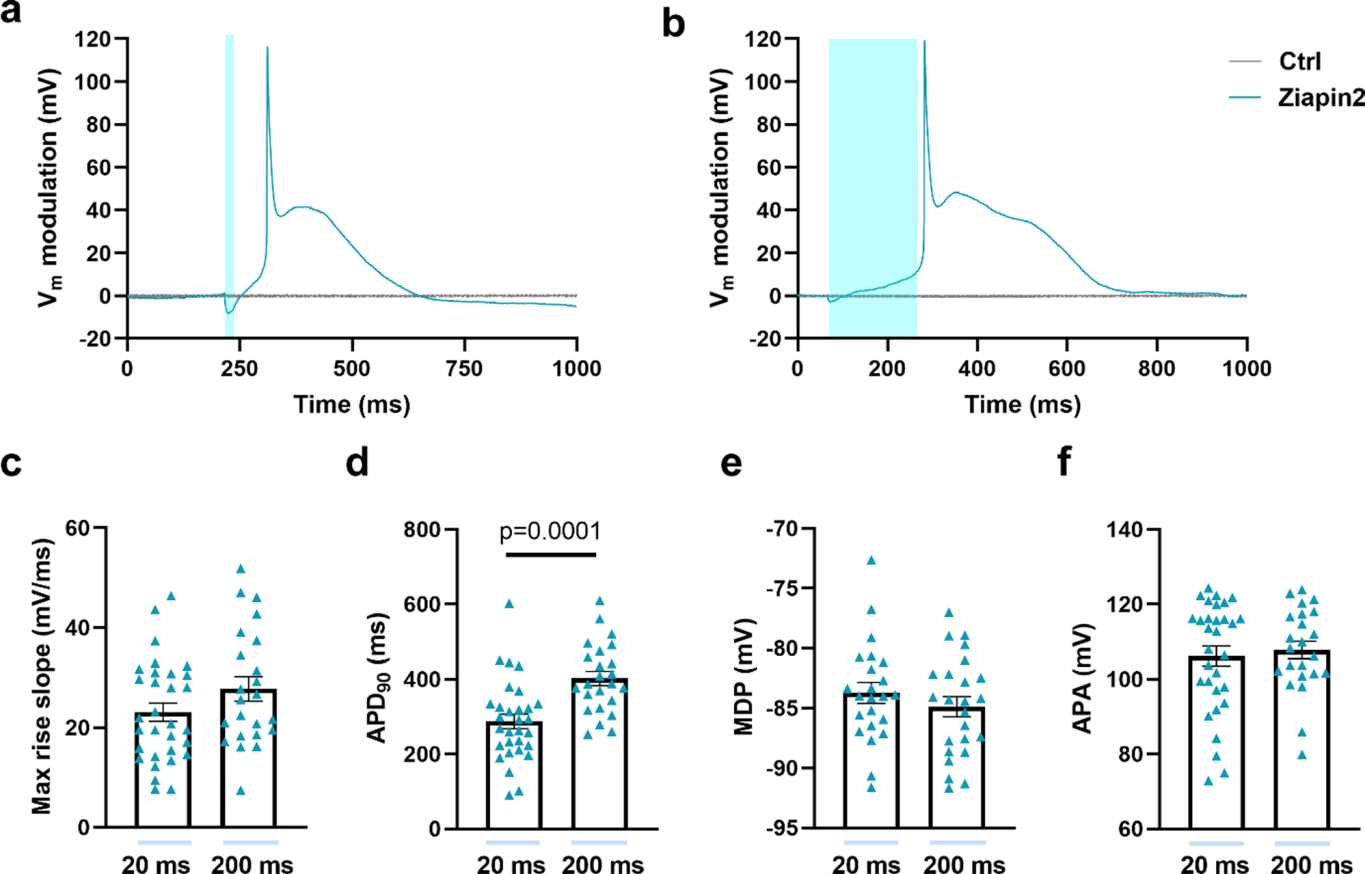
Photoinduced action potentials in Ziapin2-loaded AMVMs. Representative action potentials (APs) recorded in AMVMs loaded with either vehicle (Ctrl, shown in gray) or 25 µM Ziapin2 (shown in teal) and stimulated with short (20 ms, panel **a**) or long (200 ms, panel **b**) single light pulses. Traces have been reported as relative V_m_ variation to better appreciate the light-induced effect; photoexcitation is represented by the cyan shaded area. Light power density was set at 50 mW/mm^2^. Panels from c to f show a comparison of the following parameters between 20 and 200 ms long light-evoked APs: Maximal rise slope of depolarization (panel c, Max rise slope 20 ms: 23.1 ± 1.8 mV/ms, *n* = 31; Max rise slope 200 ms: 27.7 ± 2.4 mV/ms, *n* = 23), action potential duration at 90% of repolarization (panel d, APD90 20 ms: 287.4 ± 19.6 ms, *n* = 30; APD90 200 ms: 402 ± 19 ms, *n* = 24), maximum diastolic potential (panel e, MDP 20 ms: -83.7 ± 0.9 mV, *n* = 23; MDP 200 ms: -84.88 ± 0.8 mV, *n* = 24) and action potential amplitude (panel f, APA 20 ms: 106.1 ± 2.6 mV, *n* = 31; APA 200 ms: 107.8 ± 2.3 mV, *n* = 23). Data are represented as mean ± SEM; *N* = 10. Statistical comparisons were performed using either the Student’s t-test (panels **c, d**, and **e**) or the Mann-Whitney test (panel **f**), depending on the normality of the data distribution

Interestingly, in the majority of the AMVMs analyzed (78% for 20 ms and 97% for 200 ms light stimuli), the Vm depolarization reached the threshold for the opening of Na_V_1.5 channels, leading to the generation of an action potential (Fig. 2). In agreement with previous evidence in hiPSC-CMs [19] and NRVMs [20], this also modulates the contractile behavior of the cells (Fig. S3). By exploiting a custom MATLAB code, we obtained a quantitative analysis of the highspeed movies that allowed monitoring the effect of Ziapin2 photoisomerization on the contraction rate. In dark, Ziapin2-loaded AMVMs had a contraction rate of 0.25 ± 0.04 Hz. Under 1 Hz pulsed illumination, the rate dramatically increased during the entire acquisition window (+ 228%, from 0.25 ± 0.14 Hz to 0.82 ± 0.08 Hz), while it remained constant in vehicle-treated cells subjected to the same stimulation protocol (Fig. S3).

These observations confirm that Ziapin2 is an effective light-sensitive tool for controlling cardiac cell excitability and contractility in vitro.

### Characterization of the light-induced action potentials

As the next step in the characterization process, we assessed the morphological properties of light-induced action potentials (APs) triggered by either short or long light pulses (Fig. 2a, b).

Interestingly, APs generated upon Ziapin2 photoisomerization displayed two distinct characteristics: (i) the phase 0 occurred gradually, with a slow rate of depolarization, and (ii) the action potential duration (APD) was notably prolonged compared to expectations (typically ~ 50–100 ms). To quantify the time dynamics, we measured the maximum rise slope of the depolarization phase and the APD at 90% of repolarization (APD_90_), respectively. The former was not dependent on the stimulation time (Fig. 2c), while the latter showed a + 39.8% increase between 20ms and 200ms light pulses (rising from 287.4 ± 19.6 ms to 402 ± 19 ms; Fig. 2d). Maximum diastolic potential (MDP, Fig. 2e) and AP mean amplitude (APA, Fig. 2f) were similar in both groups, proving to be independent of the duration of the optical stimulus.

To determine whether these changes were induced by the presence of the molecule itself rather than its photoisomerization, we studied electrically evoked AP in vehicle- and Ziapin2-loaded AMVMs that were not subjected to light stimulation (Fig. S4). None of the AP characteristics were altered between the two conditions, except for a slight decrease in APD_90_ in the presence of the molecule (Fig. S4d), suggesting that Ziapin2 photoisomerization was responsible for the previously observed modifications in the AP shape.

### Effect on transporters and receptors located on the sarcolemma

Since Ziapin2 in the dark dwells in the plasma membrane and reduces the membrane thickness (Fig. 1), we investigated whether this alteration in the properties of the lipid bilayer could impact the functionality of two important membrane proteins: (i) β-adrenergic receptors (β-ARs), a class of G protein-coupled receptors that regulate cardiac function in response to sympathetic nervous system stimulation, and (ii) the Sodium-Calcium Exchanger (NCX), which is essential for maintaining Ca^2+^ homeostasis [27, 28].

To test possible alterations in β-ARs function, we compared APD_90_ in vehicle- vs. Ziapin2-loaded myocytes upon administration of 100 nM Isoprenaline (Iso). Iso accelerated the repolarization consistently in all the conditions analyzed (Fig. S5a), confirming that β-ARs function is preserved also in presence of the molecule. Additionally, the variation in the other AP parameters was also similar (Fig. S5b-d), further supporting the evidence of β-ARs similar function across the two different conditions.

As for NCX, 10 mM caffeine was employed to short-circuit SR transport, and the exponential decay time constant (τdecay) of the caffeine-induced current was determined through mono-exponential curve fitting, reflecting the contribution of NCX to diastolic Ca^2+^ clearance (Fig. S6a). τdecay in Ziapin2-loaded voltage-clamped AMVMs was comparable to that of controls (Fig. S6b). Furthermore, given that a downregulation of NCX could account for the APD prolongation observed in light-evoked APs, we assessed NCX functionality during light stimulation. In this context, Ziapin2 photoisomerization did not produce any discernible effect on NCX functionality (Fig. S6a, b). Additionally, the SR Ca^2+^ content (CaSR) remained unchanged (Fig. S6c), indicating that the balance between Ca^2+^ influx and efflux was maintained under all experimental conditions.

### Biophysical mechanism responsible for the generation of light-induced action potentials

To uncover the biophysical mechanism underlying the generation of light-evoked APs, we explored the contribution of channels whose opening probability is directly influenced by Ziapin2-induced membrane perturbations (i.e., hyperpolarization-activated cyclic nucleotide–gated channels and stretch-activated channels).

#### Hyperpolarization-activated cyclic nucleotide–gated channels

**Fig. 3:**
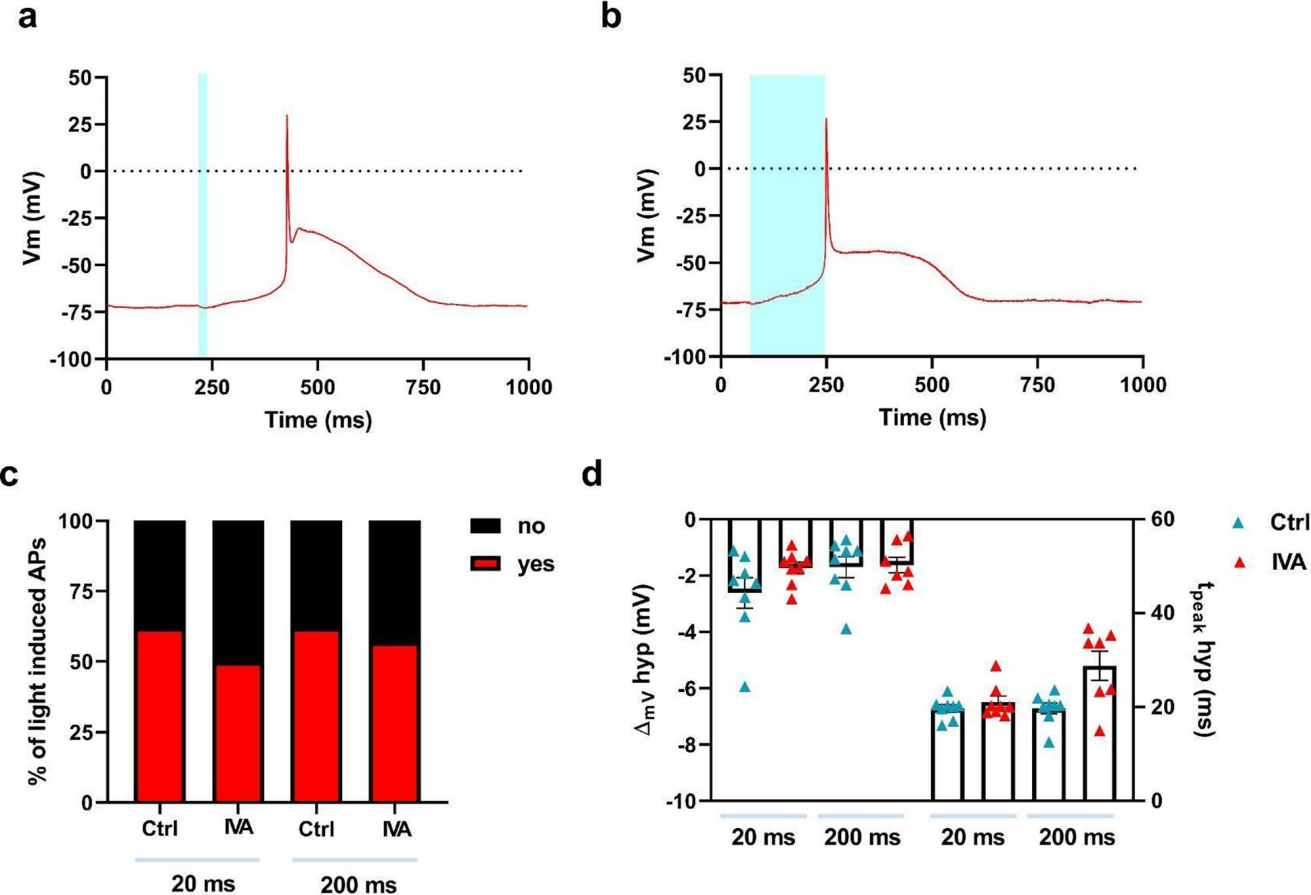
HCN channel blockade by Ivabradine does not affect light-induced action potentials in AMVMs. Representative action potentials recorded in Ziapin2-loaded cells treated with Ivabradine 10 µM (IVA) and stimulated with 20 ms- (panel **a**) or 200 ms-long (panel **b**) single light pulses. Photoexcitation is represented by the cyan shaded area. Light power density was set at 50 mW/mm^2^. **c**) Percentage of light-induced APs in Ziapin2-loaded AMVMs before and after treatment with IVA. d) Scatter plot of the peak hyperpolarization (ΔVm hyp Ctrl 20 ms: -2.62 ± 0.54 mV, *n* = 8; ΔVm hyp IVA 20 ms: -1.74 ± 0.21 mV, *n* = 8; ΔVm hyp Ctrl 200 ms: -1.7 ± 0.37 mV, *n* = 8; ΔVm hyp IVA 200 ms: -1.63 ± 0.28 mV, *n* = 7) and time-to-peak of hyperpolarization (t_peak_ hyp Ctrl 20 ms: 19.7 ± 0.79 ms, *n* = 8; t_peak_ hyp IVA 20 ms: 21.1 ± 1.2 ms, *n* = 8; t_peak_ hyp Ctrl 200 ms: 19.7 ± 1.17 ms, *n* = 8; t_peak_ hyp IVA 200 ms: 28.79 ± 3 ms, *n* = 7) in AMVMs exposed to 25 µM Ziapin2 for the above-mentioned light-stimulation protocols. Data are represented as mean ± SEM; *N* = 2. Statistical comparisons were performed using the chi-square test (panel **c**) and the Kruskal-Wallis test (panel **d**)

Hyperpolarization-activated cyclic nucleotide–gated (HCN) channels mediate the influx of I_f_, a mixed Na^+^ and K^+^ current that activates during hyperpolarization at diastolic voltages [29]. I_f_ exerts a depolarizing influence in reaching the membrane potential threshold for AP firing. To evaluate its potential role in the light-induced triggering process we treated AMVMs with Ivabradine 10 µM, a specific blocker of HCN channels [30]. The percentage of light-evoked APs was unaffected by the presence of Ivabradine, regardless of whether short or long light pulses were used (Fig. 3a-c). Notably, the magnitude of the hyperpolarization and the time-to-peak (Fig. 3d) were unaltered. Overall, these data suggest that HCN channels do not contribute to the genesis of light-induced AP.

#### Stretch-activated channels

Stretch-activated channels (SACs) are ion channels found in cell membranes that open or close in response to mechanical deformation or stretching of the membrane [31]. SACs could be subdivided by their ion selectivity into K^+^-selective (SAC_K_) and cation non-selective channels (SAC_NS_).

Recently, it has been reported in HEK-293T cells transfected with the SAC_K_ TWIK-related arachidonic acid-stimulated K^+^ (TRAAK) that the insertion of Ziapin2 into the plasma membrane can modulate its activity [32].

**Fig. 4:**
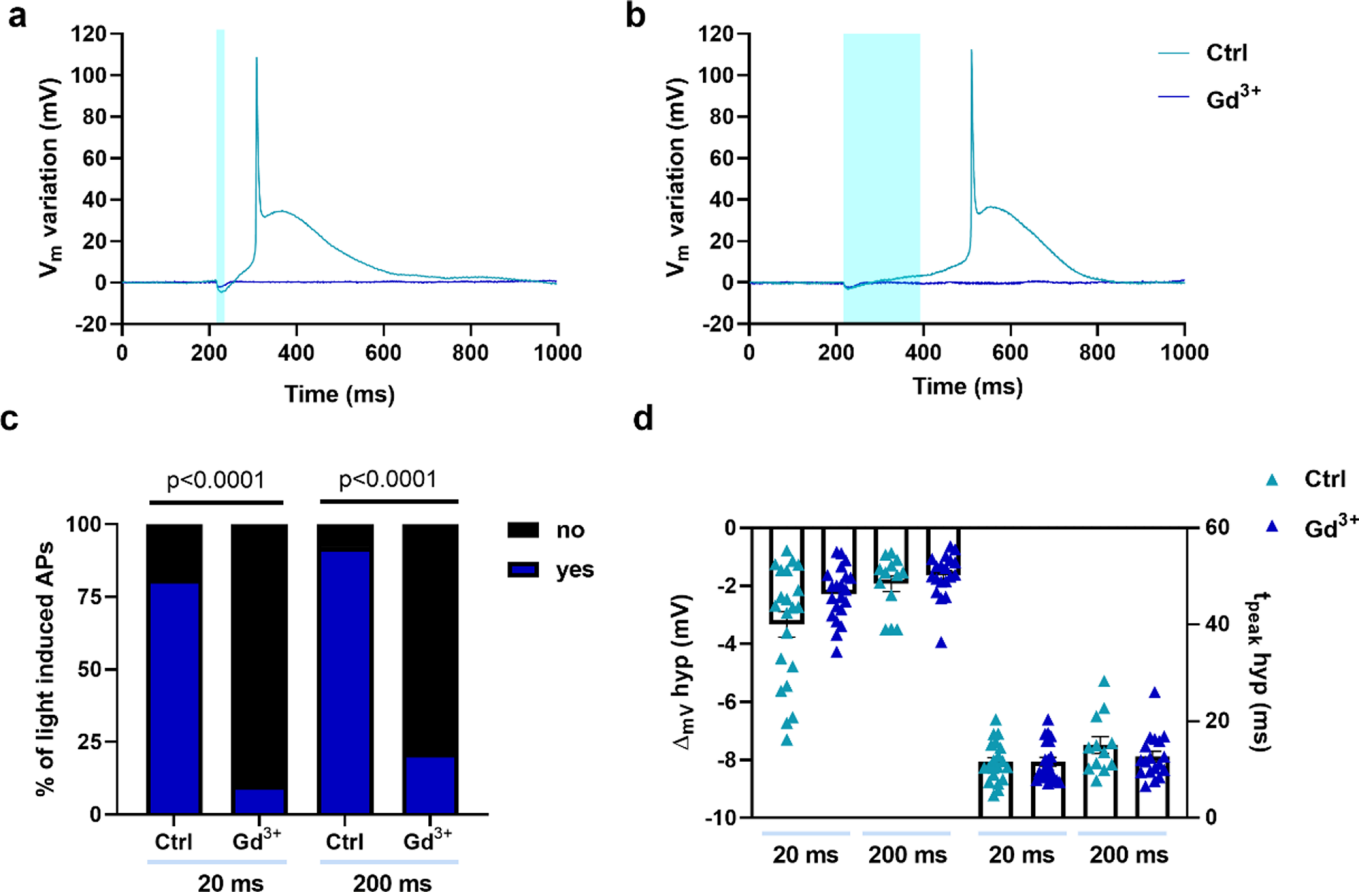
Gadolinium blockade reveals critical role of stretch-activated channels in light-induced action potential generation in AMVMs. Representative action potentials recorded from Ziapin2-loaded cells before and after treatment with Gadolinium 50 µM (Gd^3+^) and stimulated with 20 ms- (panel **a**) or 200 ms-long (panel **b**) single light pulses. Traces have been reported as relative V_m_ variation to better appreciate the light-induced effect; photoexcitation is represented by the cyan shaded area. Light power density was set at 50 mW/mm^2^. **c**) Percentage of light-induced APs in Ziapin2-loaded AMVMs before and after treatment with Gd^3+^. **d**) Scatter plot of the peak hyperpolarization (ΔVm hyp Ctrl 20 ms: -3.31 ± 0.44 mV, *n* = 21; ΔVm hyp Gd^3+^ 20 ms: -2.27 ± 0.2 mV, *n* = 21; ΔVm hyp Ctrl 200 ms: -1.9 ± 0.27 mV, *n* = 13; ΔVm hyp Gd^3+^ 200 ms: -1.63 ± 0.17 mV, *n* = 19) and time-to-peak of hyperpolarization (t_peak_ hyp Ctrl 20 ms: 11.5 ± 0.93 ms, *n* = 20; t_peak_ hyp Gd^3+^ 20 ms: 11.64 ± 0.89 ms, *n* = 21; t_peak_ hyp Ctrl 200 ms: 15 ± 1.7 ms, *n* = 12; t_peak_ hyp Gd^3+^ 200 ms: 12.68 ± 1 ms, *n* = 18) in AMVMs exposed to 25 µM Ziapin2 for the above-mentioned light-stimulation protocols. Data are represented as mean ± SEM; Ctrl: *N* = 3. Statistical comparisons were performed using the Fisher’s exact test (panel **c**) and the Kruskal Wallis test (panel **d**)

To investigate the potential involvement of SACs in AMVMs AP generation, Ziapin2-loaded cells were treated with Gadolinium (Gd^3+^, 50 µM), a non-specific blocker of these channels [33, 34]. In the presence of Gd^3+^, the magnitude and the time-to-peak of the hyperpolarization phase were unchanged (Fig. 4d), while the percentage of 20 and 200 ms light-evoked APs was dramatically reduced by 88% and 77%, respectively (Fig. 4a-c), suggesting a pivotal role of SACs in the triggering process.

**Fig. 5:**
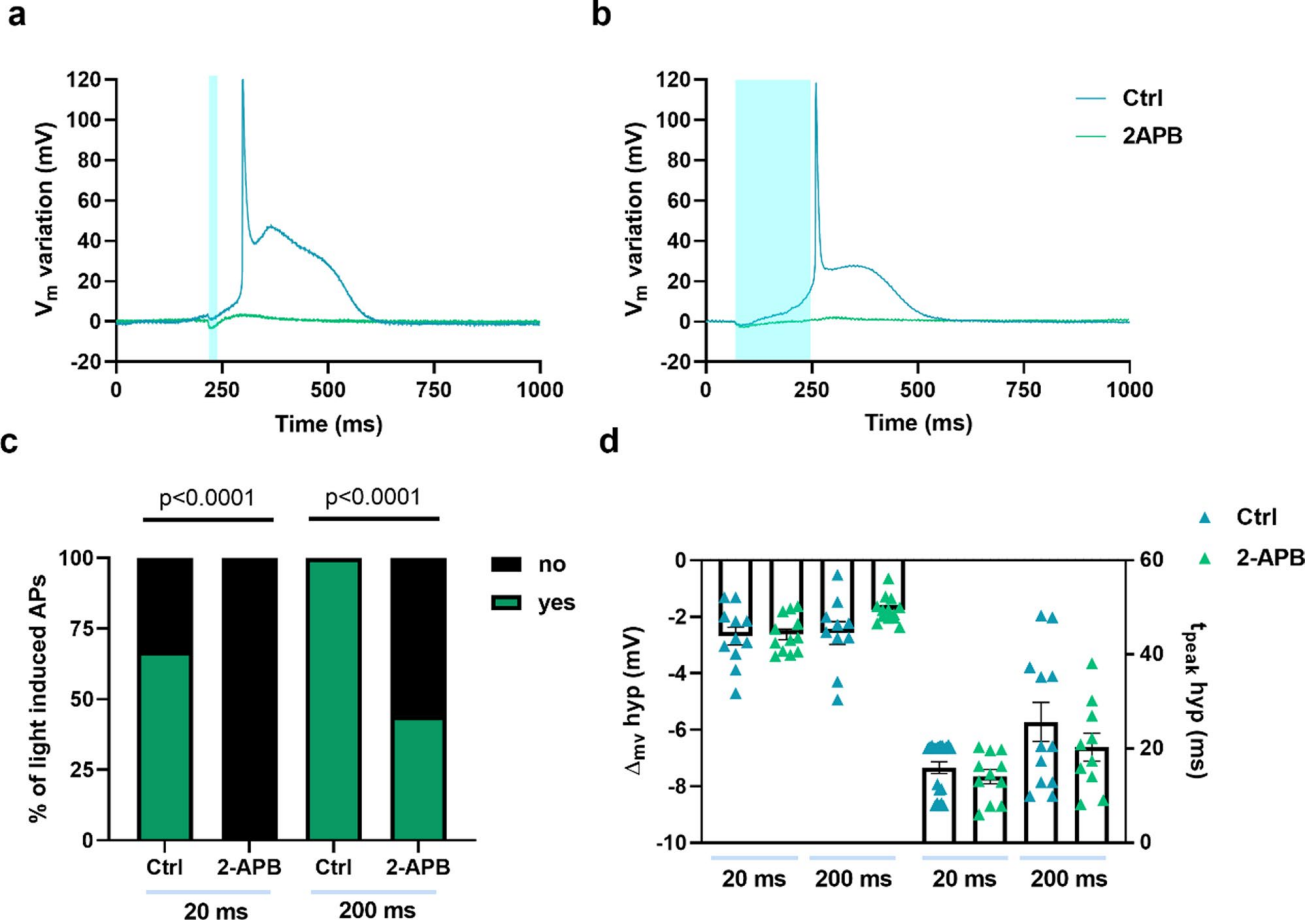
Effect of 2-APB on light-induced action potential generation highlights the role of SACNS. Representative action potentials recorded from Ziapin2-loaded cells before and after treatment with 2-Aminoethyl Diphenylborinate 75 µM (2-APB) and stimulated with 20 ms- (panel **a**) or 200 ms-long (panel **b**) single light pulses. Traces have been reported as relative V_m_ variation to better appreciate the light-induced effect; photoexcitation is represented by the cyan shaded area. Light power density was set at 50 mW/mm^2^. **c**) Percentage of light-induced APs in Ziapin2-loaded AMVMs before and after treatment with 2-APB. **d**) Scatter plot of the peak hyperpolarization (ΔVm hyp Ctrl 20 ms: -2.68 ± 0.31 mV, *n* = 11; ΔVm hyp 2-APB 20 ms: -2.62 ± 0.19 mV, *n* = 12; ΔVm hyp Ctrl 200 ms: -2.57 ± 0.4 mV, *n* = 10; ΔVm hyp 2-APB 200 ms: -1.74 ± 0.12 mV, *n* = 13) and time-to-peak of hyperpolarization (t_peak_ hyp Ctrl 20 ms: 15.9 ± 1.2 ms, *n* = 20; t_peak_ hyp 2-APB 20 ms: 14.6 ± 1.5 ms, *n* = 11; t_peak_ hyp Ctrl 200 ms: 25.6 ± 4.1 ms, *n* = 12; t_peak_ hyp 2-APB 200 ms: 20.3 ± 2.9 ms, *n* = 10) in AMVMs exposed to 25 µM Ziapin2 for the above-mentioned light-stimulation protocols. Data are represented as mean ± SEM; Ctrl: *N* = 4. Statistical comparisons were performed using the Fisher’s exact test (panel **c**) and the ordinary one-way ANOVA test (panel **d**)

However, Gd^3+^ acts as a generic blocker, preventing the distinction between SAC_K_ and SAC_NS_. Several SAC_NS_ play key roles in the heart, among which different subtypes of Transient Receptor Potential (TRP) channels, such as TRPC (canonical), TRPV (vanilloid), and TRPM (melastatin) channels, that primarily facilitates the movement of Ca²⁺, Na⁺, and to a lesser extent, K⁺ ions across the cell membrane [31]. To further investigate the involvement of SAC_NS_, AMVMs were treated with the TRP blocker 2-Aminoethyl diphenylborinate (2-APB, 75 µM) [35–37]. Superfusion with 2-APB sharply lowered the light-mediated firing probability for both stimulation durations (Fig. 5a-c), highlighting the critical role of TRP SAC_NS_. Despite this effect, neither the hyperpolarization amplitude nor the time to peak was altered (Fig. 5d).

Remarkably, both Gd^3+^ and 2-APB treatments preserved the biphasic Vm modulation observed in non-excitable cells [15, 16, 18, 32], featuring a fast hyperpolarization (Figs. 4d and 5d) followed by a slight delayed depolarization of approximately 1.5 mV (Fig. S7, S8). This finding confirms that the initial phenomenon, driven by Ziapin2 photoisomerization, is exclusively attributable to a capacitive effect (Scheme 1).

**Fig. 6:**
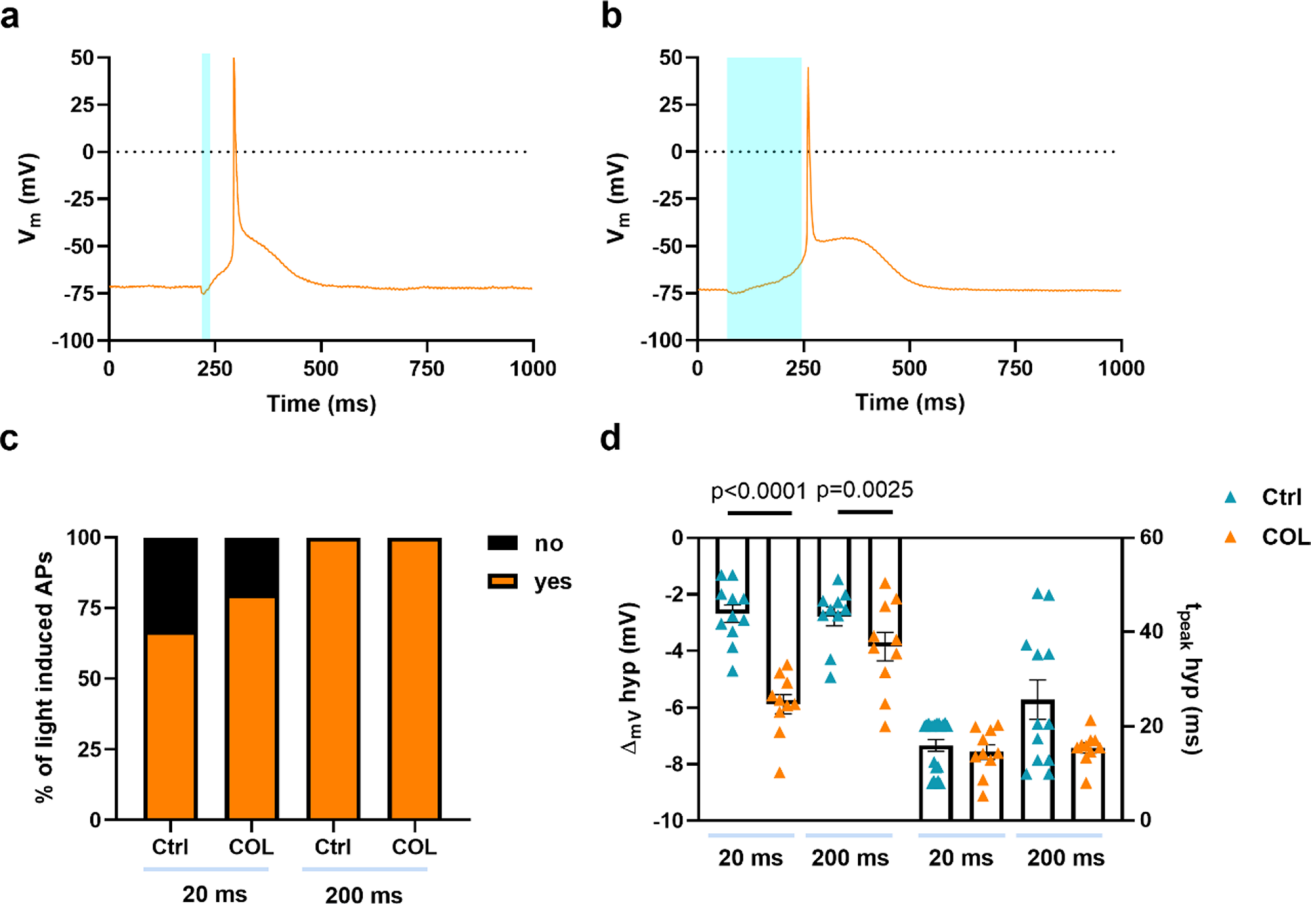
Disruption of microtubules did not impact the efficiency of light-evoked action potential generation in AMVMs. Representative action potentials recorded in Ziapin2-loaded cells treated with Colchicine 10 µM (COL) and stimulated with 20 ms- (panel **a**) or 200 ms-long (panel **b**) single light pulses. Photoexcitation is represented by the cyan shaded area. Light power density was set at 50 mW/mm^2^. **c**) Percentage of light-induced APs in Ziapin2-loaded AMVMs before and after treatment with COL. d) Scatter plot of the peak hyperpolarization (ΔVm hyp Ctrl 20 ms: -2.68 ± 0.32 mV, *n* = 11; ΔVm hyp COL 20 ms: -5.87 ± 0.34 mV, *n* = 10; ΔVm hyp Ctrl 200 ms: -2.77 ± 0.33 mV, *n* = 10; ΔVm hyp COL 200 ms: -3.85 ± 0.5 mV, *n* = 10) and time-to-peak of hyperpolarization (t_peak_ hyp Ctrl 20 ms: 15.9 ± 1.2 ms, *n* = 20; t_peak_ hyp COL 20 ms: 14.57 ± 1.54 ms, *n* = 10; t_peak_ hyp Ctrl 200 ms: 25.67 ± 4.1 ms, *n* = 12; t_peak_ hyp COL 200 ms: 15.46 ± 1 ms, *n* = 10) in AMVMs exposed to 25 µM Ziapin2 for the above-mentioned light-stimulation protocols. Data are represented as mean ± SEM; Ctrl: *N* = 2, COL: *N* = 2. Statistical comparisons were performed using the Fisher’s exact test (panel **c**) and ordinary one-way ANOVA (panel **d**)

Finally, since in various eukaryotic cells SACs are thought to be functionally and structurally coupled to the cytoskeleton, we studied the impact of cytoskeleton disassembly. To this extend, Ziapin2-loaded AMVMs were pre-incubated with Colchicine 10 µM [38] and subjected to short and long light pulses (Fig. 6a, b). Microtubule disruption did not affect the efficiency of light-evoked APs generation (Fig. 6a-c), suggesting that SAC_NS_ respond directly to mechanical stimuli rather than through interactions with cytoskeletal components. Surprisingly, cells pre-incubated with Colchicine exhibited significantly higher hyperpolarization at both light-stimulus durations, while the time-to-peak was unchanged across the different conditions (Fig. 6d). This could be explained by the fact that Colchicine-induced disruption of microtubules might alter the distribution of mechanical forces within the cell, potentially leading to changes in membrane tension and increased stretch.

To summarize, our data hint that SACs, and particularly SAC_NS_, contribute to the light-induced triggering process mediated by Ziapin2.

#### Proposed mechanism of interaction between Ziapin2 and SACs in AMVMs

SACs open in the dark, when Ziapin2 molecules, anchored to the opposite leaflets of the sarcolemma, trans-dimerize causing a local depression of the membrane (Scheme 1).

Considering this, one might expect a modulation of the resting Vm under basal conditions; however, in our experiments, we did not observe any change (Fig. 2e). This could be explained by the cooperative presence of both SAC_K_ and SAC_NS_, which might counterbalance each other’s effect allowing cells to reach a novel equilibrium. Furthermore, AMVMs express inwardly rectifying potassium channels, which are crucial for maintaining the resting Vm, even during ionic imbalances or changes in extracellular K^+^ concentrations [39].

When photostimulation is applied, Ziapin2 undergoes a conformational change (from trans→cis), resulting in membrane relaxation and subsequent closure of SACs [32]. Simultaneously, the variation of membrane capacitance, due to Ziapin2 isomerization, accounts for the biphasic Vm modulation (Scheme 1), as confirmed by our experiments in presence of SACs blockers (Fig. S7, S8). Upon light offset, the molecules, or at least a fraction of them, will return to the trans conformation, thereby stretching the plasma membrane and re-opening the SACs. This will allow the influx of a depolarizing current necessary to reach the threshold for sodium channel activation. This current will persist for several milliseconds, contributing to the longer duration of light-induced APs (Fig. 2). Interestingly, the duration of the APs under 200 ms light stimulation was longer (+ 39.8%) compared to 20 ms light pulses (Fig. 2d). We can speculate that longer pulses recruit more molecules, which affects the overall time required for the trans→cis conversion.

Finally, since our experiments suggest a prominent contribution of TRP SAC_NS_, we hypothesized that a Ca^2+^ conductance might be involved. To test this, we reduced the extracellular [Ca^2+^] to 1.2 mM. Under these conditions, light-evoked APs showed a notably longer APD_90_ compared to controls (Fig. S9), but had a shorter duration than those recorded in the presence of 2mM [Ca^2+^] (Fig. S9d). This implies that Ca^2+^ is important in shaping AP characteristics, supporting the hypothesis that Ca^2+^ influx through SAC_NS_ might be crucial to the whole process.

## Conclusions

Ziapin2 represents a significant advancement in the field of cardiac cell modulation, offering a precise, non-invasive, and safe tool for controlling the ECC. By eliminating the need for genetic manipulation and the associated risks of introducing exogenous genetic material, Ziapin2 holds great promise as a light-sensitive transducer for both research and clinical applications. However, to fully realize the potential of Ziapin2 across a wider range of applications, it is essential to elucidate its biophysical mechanisms of action. This study addresses this critical aspect, highlighting the role of SACs as key mediators in translating the mechanical perturbations induced by Ziapin2 into electrical signals. Our experiments support the evidence that Ca^2+^ influx via SAC_NS_ may be the principal biological determinant of the light-induced AP generation process, also contributing to the observed APD prolongation. 2-APB has been shown to block TRP channels, nevertheless, the role of these channels in stretch activation is complex and likely context dependent. In that regard, TRPC channels, particularly TRPC1 and TRPC6, are the most studied in the context of stretch-activated responses [40, 41]. However, not all studies agree on the stretch-activated nature of TRP channels. Some research has failed to replicate the stretch activation of TRP channels or has found conflicting results [42]. Furthermore, we cannot entirely exclude the contribution of Piezo1 channels, which may also play a role in the observed effects.

Given the complexity of the biological framework and the absence of truly specific blockers, we aim to further corroborate our findings by developing a computational model of murine APs that incorporates both the variation in membrane capacitance resulting from Ziapin2 isomerization and SACs.

Overall, this work fosters a deeper understanding of the phenomena underlying the Ziapin2-mediated photostimulation process in cardiac excitable cells, thereby opening interesting prospects for its application in cardiovascular research.

## Electronic supplementary material

Below is the link to the electronic supplementary material.

Supplementary Material 1

## Data Availability

The datasets used and/or analyzed during the current study are available from the corresponding author on reasonable request.

## Abbreviations

2-APB: 2-Aminoethyl diphenylborinate
AMVMs: Adult mouse ventricular myocytes
AP: Action potential
APA: Action potential amplitude
APD: Action potential duration
APD_90_: Action potential duration at 90% of repolarization
CaSR: SR Ca^2+^ content
ECC: Excitation-contraction coupling
Gd^3+^: Gadolinium
hiPSC-CMs: Human-induced pluripotent stem cells–derived cardiomyocytes
Iso: Isoprenaline
LED: Light-emitting diode
MDP: Maximum diastolic potential
NCX: Sodium-calcium exchanger
NRVMs: Neonatal rat ventricular myocytes
ROI: Region of interest
SAC_K_: K^+^-selective stretch-activated channels
SAC_NS_: Cation non-selective stretch-activated channels
SACs: Stretch-activated channels
SR: Sarcoplasmic reticulum
t_peak_: Time to peak
TRAAK: TWIK-related arachidonic acid-stimulated K^+^ channel
TRP: Transient Receptor Potential
Vm: Membrane potential
β-AR: β-adrenergic receptor
