# Supplementary material for: Role of stretch-activated channels in light-generated action potentials mediated by an intramembrane molecular photoswitch
Source: J Transl Med. 2024 Nov 27;22:1068. doi: 10.1186/s12967-024-05902-4 (PMC11600573; doi:10.1186/s12967-024-05902-4)
Supplement: Supplementary file 1 — Supplementary Material 1 [file 12967_2024_5902_MOESM1_ESM.docx]

**SUPPLEMENTARY FIGURES**

**Role of stretch-activated channels in light-generated action potentials mediated by an intramembrane molecular photoswitch**

Chiara Florindi^1,2^, Vito Vurro^2^, Paola Moretti^2,3^, Chiara Bertarelli^2,3^, Antonio Zaza^1^, Guglielmo Lanzani^2,4^, Francesco Lodola^1,2*^

^1^Department of Biotechnology and Biosciences, University of Milano-Bicocca, Milan, Italy;

^2^Center for Nano Science and Technology, Istituto Italiano di Tecnologia, Milan, Italy;

^3^Department of Chemistry, Materials and Chemical Engineering “Giulio Natta”, Politecnico di Milano, Milan, Italy;

^4^Department of Physics, Politecnico di Milano, Milan, Italy.


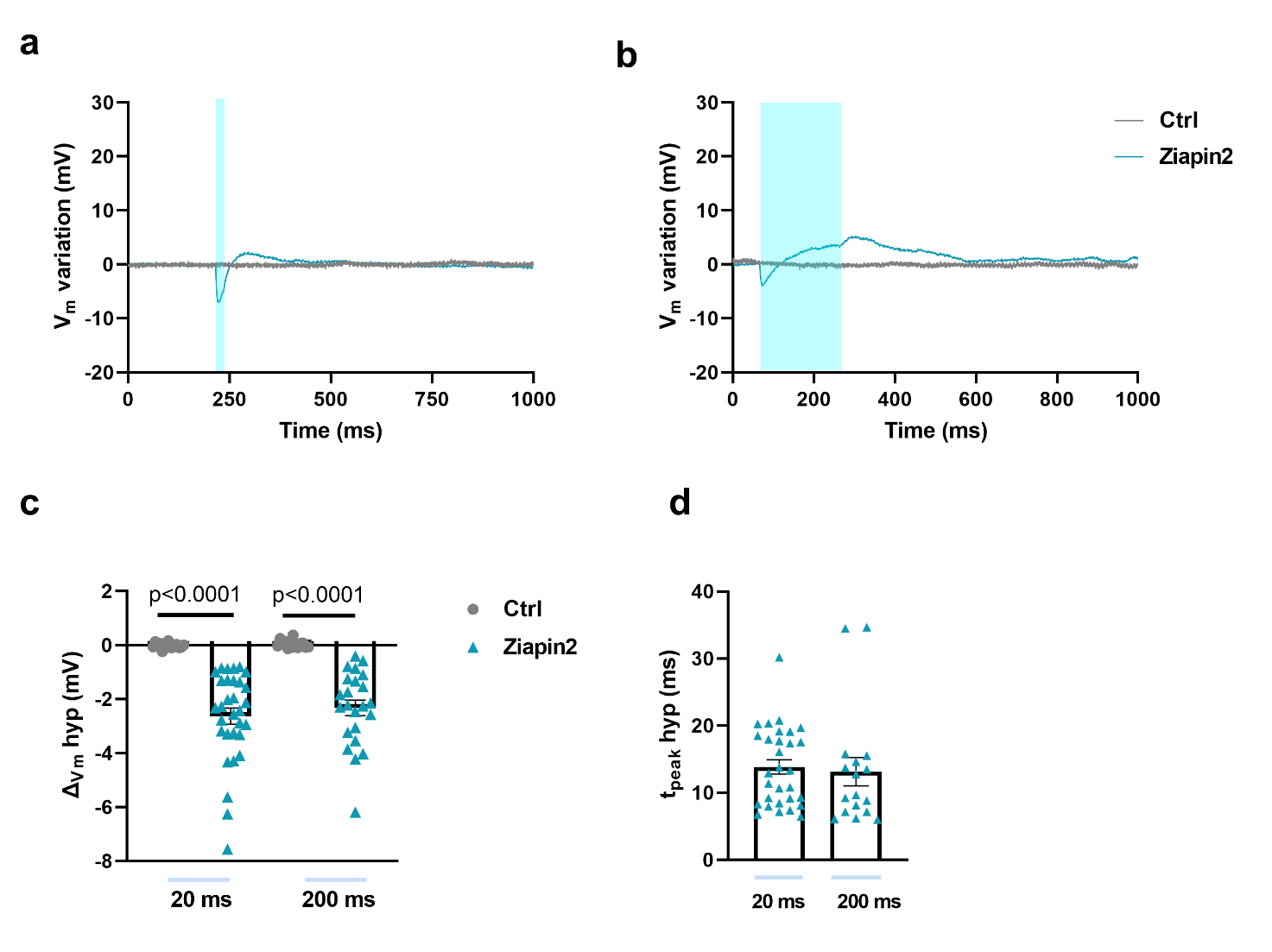


***Figure S1: Ziapin2 mediates a light-evoked membrane voltage modulation in AMVMs.*** *Representative whole-cell current-clamp traces recorded in cells loaded with either vehicle (Ctrl, in gray) or Ziapin2 (teal) and stimulated with 20 ms- (panel a) or 200 ms-long (panel b) single light pulses.* *Traces have been reported as relative V_m_ variation to better appreciate the light-induced effect; photoexcitation is represented by the cyan shaded area. Light power density was set at 50 mW/mm^2^. Scatterplot of the peak hyperpolarization (panel c, Δ_Vm_ hyp Ctrl 20 ms: -0.01 ± 0.02 mV, n = 15; Δ_Vm_ hyp Ziapin2 20 ms: -2.63 ± 0.29 mV, n = 31; Δ_Vm_ hyp Ctrl 200 ms: 0.04 ± 0.03 mV, n = 14; hyp Ziapin2 200 ms: -2.35 ± 0.29 mV, n = 23) and time-to-peak of hyperpolarization (panel d, t_peak_ hyp 20 ms: 13.9 ± 1 ms, n = 30; t_peak_ hyp 200 ms: 13.1 ± 2.1 ms, n = 17) in AMVMs exposed to 25 μM Ziapin2 or DMSO for the above-mentioned light-stimulation protocols. Data are represented as mean ± SEM; Ctrl: N = 2, Ziapin2: N = 9. Statistical comparisons were performed using Kruskal-Wallis test (panel c) and the Mann-Whitney test (panel d).*


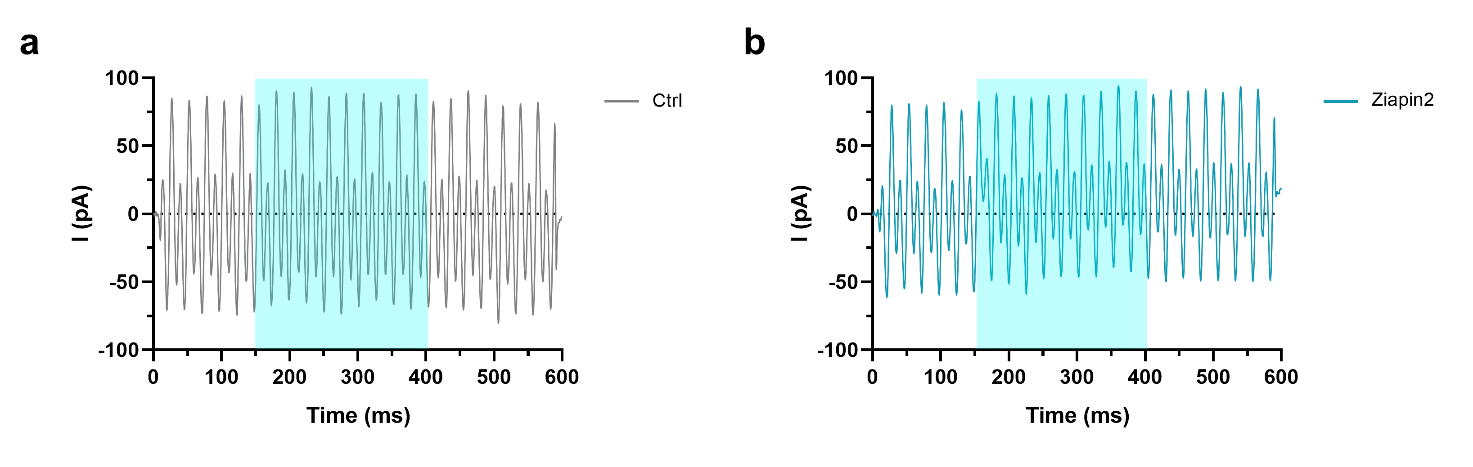
***Fig. S2: Capacitance recordings in AMVMs.*** *Representative membrane current traces recorded in AMVMs loaded with either vehicle (Ctrl, shown in grey in panel a) or 25 µM Ziapin2 (shown in teal in panel b) during voltage-clamp double sinusoidal stimulation. Recordings were taken both in dark conditions and upon 250 ms light stimulation (indicated by the cyan shaded area) to assess Ziapin2-mediated changes in capacitance. Light power density was set at 50 mW/mm².*


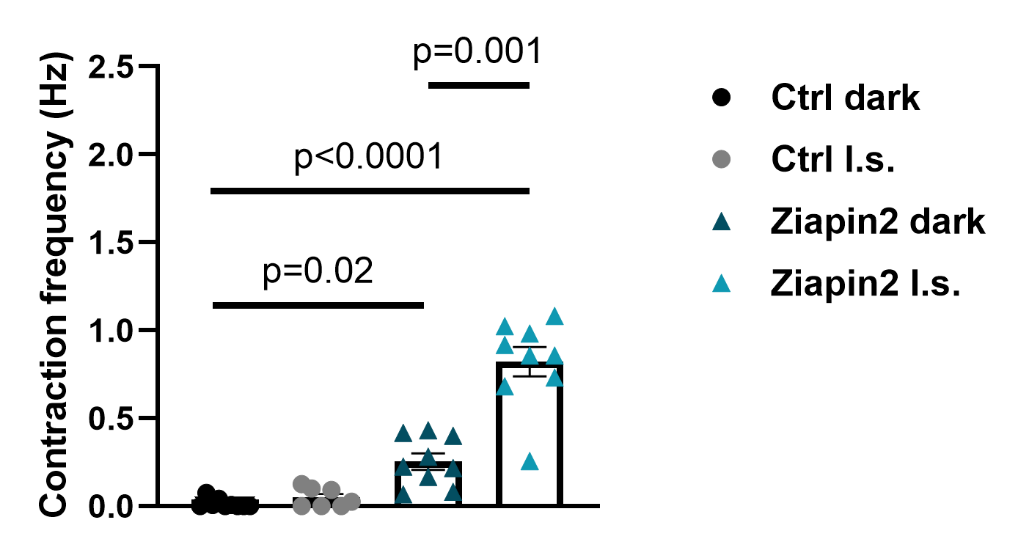


***Figure S3: Light-induced contraction rate modulation in Ziapin2 loaded AMVMs.*** *Vehicle or Ziapin2-treated AMVMs contraction frequency before and after 1 Hz pulsed light stimulation with a light power density set at 30 mW/mm^2^. The experiments were carried out at room temperature (24°C). Ctrl dark: 0.01 ± 0.02 Hz, n = 9; Ctrl l.s: 0.04 ± 0.05 Hz, n = 7; Ziapin2 dark: 0.25 ± 0.14 Hz, n = 9; Ziapin2 l.s: 0.82 ± 0.25 Hz, n = 9. Data are shown as mean ± SEM. Ctrl: N = 2; Ziapin2: N = 2. Multiple comparisons were performed using the Kruskal-Wallis test.*


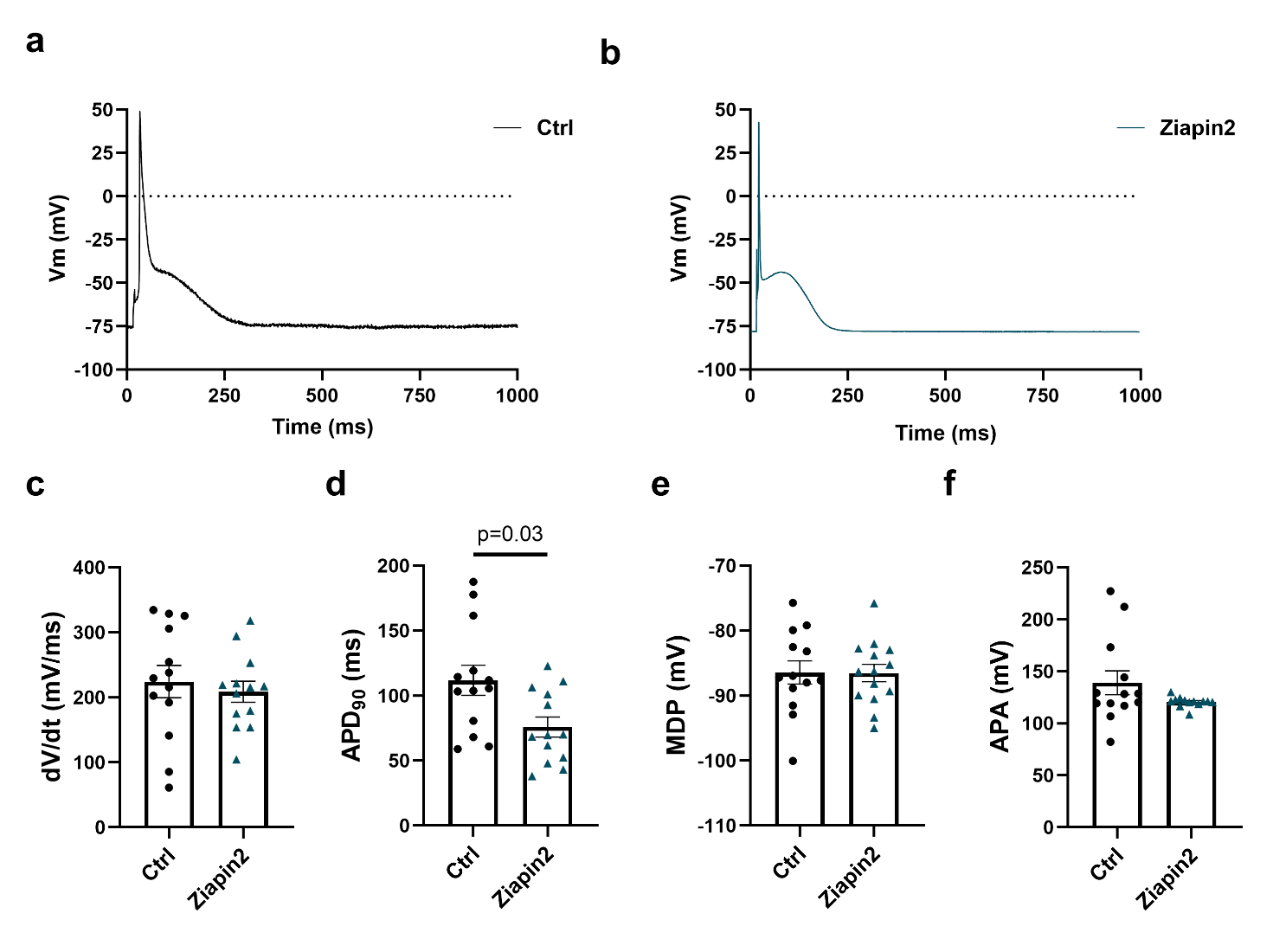


***Figure S4: Effect of Ziapin2 on electrically evoked action potentials.*** *Representative electrically induced action potentials (APs) recorded in AMVMs loaded with either vehicle (Ctrl, panel a) or 25 µM Ziapin2 (Ziapin2, panel b). Panels from c to f show a comparison of the following parameters between 20 and 200 ms long light-evoked APs: Maximum action potential upstroke velocity (panel c, dV/dt Ctrl: 224.2 ± 24.7 mV/ms, n = 13; dV/dt Ziapin2: 208.6 ± 16.3 mV/ms, n = 13), action potential duration at 90% of repolarization (panel d, APD_90_ Ctrl: 111.8 ± 11.6 ms, n = 13; APD_90_ Ziapin2: 75.8 ± 7.79 ms, n = 13), maximum diastolic potential (panel e, MDP Ctrl: -86.4 ± 1.8 mV, n = 13; MDP Ziapin2: -86.5 ± 1.3 mV, n = 14) and action potential amplitude (panel f, APA Ctrl: 139.1 ± 11.5 mV, n = 13; APA Ziapin2: 120.7 ± 1.3 mV, n = 14). Data are represented as mean ± SEM; Ctrl: N = 4; Ziapin2: N = 3. Statistical comparisons were performed using the Mann-Whitney test.*


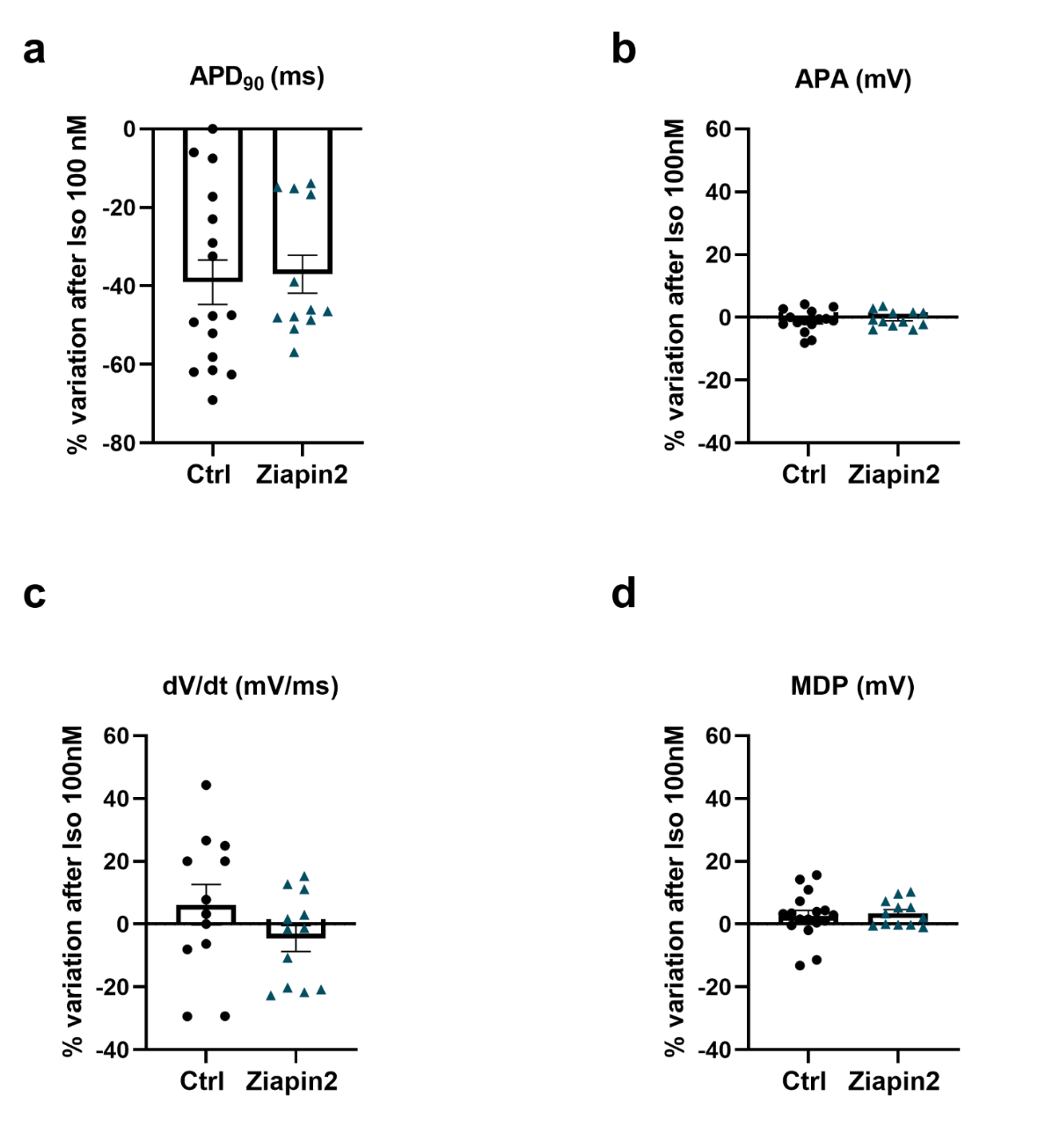


***Figure S5: AP parameters in AMVMs upon β-adrenergic stimulation.*** *Comparison of the action potential duration at 90% of repolarization (panel a, Δ% APD_90_ Ctrl: -39 ± 5.7, n = 16; Δ% APD_90_ Ziapin2: -37 ± 4.8, n = 12), action potential amplitude (panel b, Δ% APA Ctrl: -1.12 ± 0.9, n = 15; Δ% APA Ziapin2: -0.40 ± 0.7, n = 12), maximum action potential upstroke velocity (panel c, Δ% dV/dt Ctrl: 6.1 ± 6.5, n = 12; Δ% dV/dt Ziapin2: -4.6 ± 4.1, n = 12) and maximum diastolic potential (panel d, Δ% Ctrl MDP: 2.5 ± 1.8, n = 17; Δ% MDP Ziapin2: 3.46 ± 1.1, n = 12) between electrically evoked APs loaded with either vehicle (Ctrl) or 25 µM Ziapin2 and exposed to 100 nM Isoprenaline (Iso). Data are represented as mean ± SEM; Ctrl: N = 5; Ziapin2: N = 2. Statistical comparisons were performed with either the Mann-Whitney test (panels a, b, and d) or the Welch’s t-test (panel c), depending on the normality of the data distribution.*

**
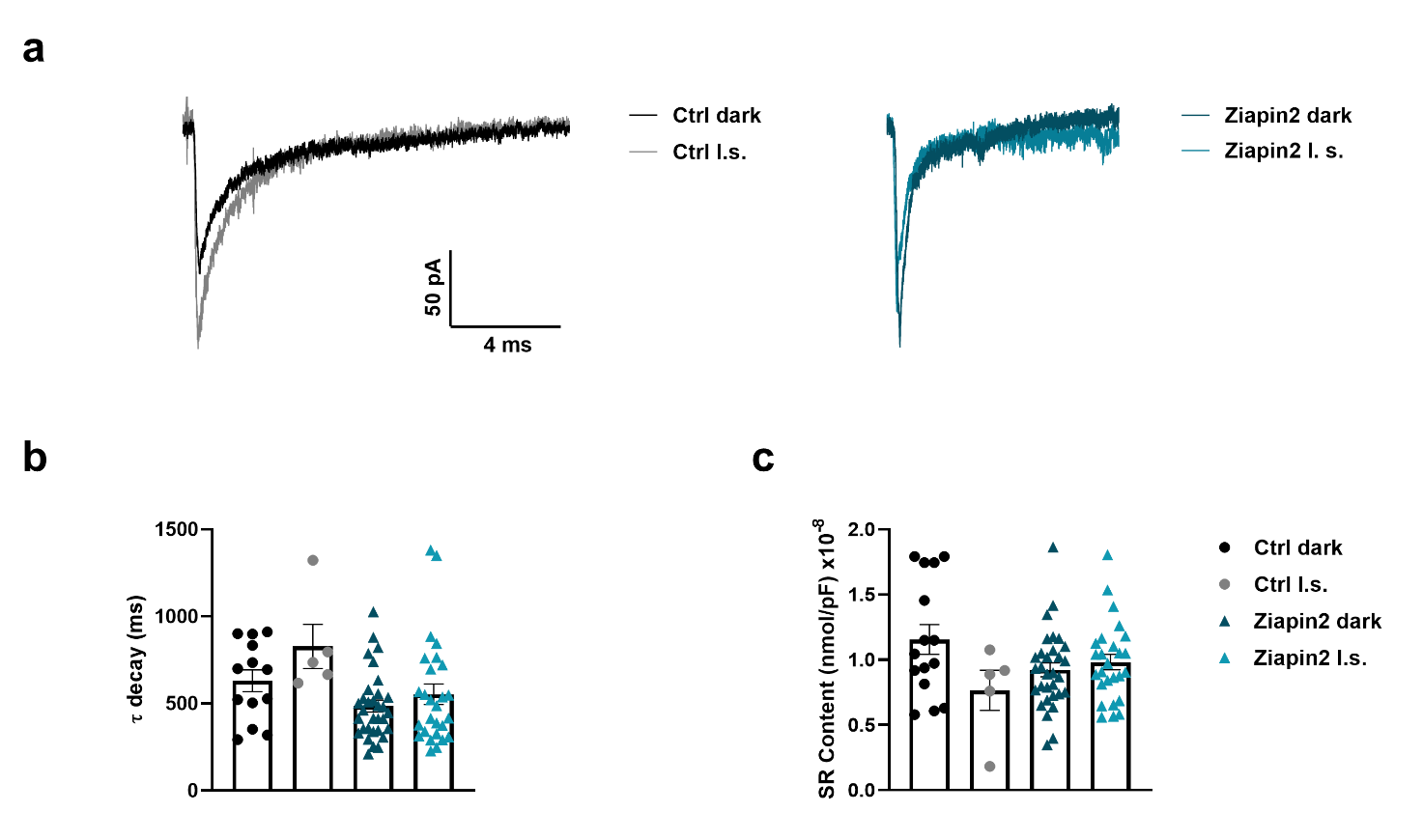
*Figure S6: Ziapin2 does not alters NCX function in both dark and light-conditions****. a) Representative caffeine-induced NCX currents recorded in vehicle (Ctrl, left panel) or 25 µM Ziapin2-loaded (right panel) AMVMs acquired in dark conditions or upon photostimulation at a light power density of 50 mW/mm^2^. Tau of fast component of I_NCX_ decay (panel b, Ctrl dark: 629.5 ± 62.6 ms, n = 13; Ctrl l.s.: 827.1 ± 127.4 ms, n = 5; Ziapin2 dark: 485 ± 34 ms, n = 32; Ziapin2 l.s.: 552.4 ± 57.7 ms, n = 27) and SR content (panel c, Ctrl dark: 1.15 ± 0.11 x 10^-8^ nmol/pF, n = 15; Ctrl l.s.: 0.76 ± 0.15 x 10^-8^ nmol/pF, n = 5; Ziapin2 dark: 0.92 ± 0.05 x 10^-8^ nmol/pF, n = 31; Ziapin2 l.s.: 0.98 ± 0.06 ms, n = 26) among the different experimental conditions. Data are represented as mean ± SEM. Ctrl dark N = 5; Ctrl l.s. N= 1; Ziapin2 dark N = 5; Ziapin2 l.s. N= 3. Statistical comparisons were performed with either the Kruskal Wallis test (panel c) or the ordinary one-way ANOVA (panel d), depending on the normality of the data distribution.*


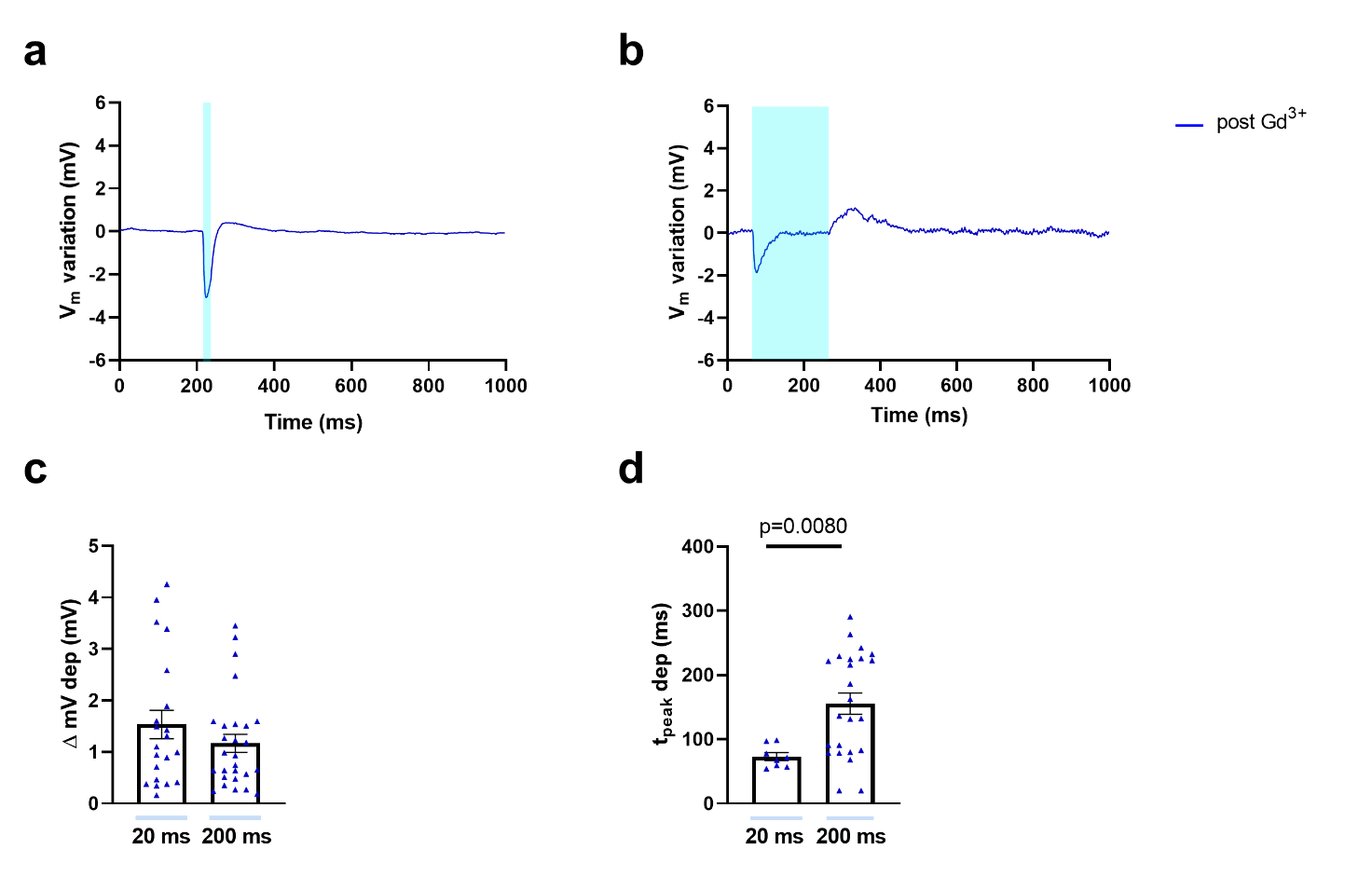


***Figure S7: V_m_ depolarization is preserved in light-stimulated Ziapin2-loaded AMVMs upon treatment with Gd^3+^.*** *Representative whole-cell current-clamp traces recorded in cells loaded with Ziapin2 and stimulated with 20 ms- (panel a) or 200 ms-long (panel b) single light pulses, following 50 μM Gd³⁺ treatment. Traces have been reported as relative V_m_ variation to better appreciate the light-induced effect; photoexcitation is represented by the cyan shaded area. Light power density was set at 50 mW/mm^2^. Peak depolarization (panel c, Δ_Vm_ dep 20 ms: 1.53 ± 0.27 mV, n = 21; Δ_Vm_ dep 200 ms: 1.25 ± 0.25 mV, n = 18) and time-to-peak of depolatization (panel d, t_peak_ dep 20 ms: 72.9 ± 6.11 ms, n = 8; t_peak_ dep 200 ms: 192 ± 15.3 ms, n = 17) in 25 μM Ziapin2-loaded AMVMs exposed to 50 μM Gd^3+^. Data are represented as mean ± SEM; N = 3.* *Statistical comparison was performed with the Mann-Whitney test.*

***
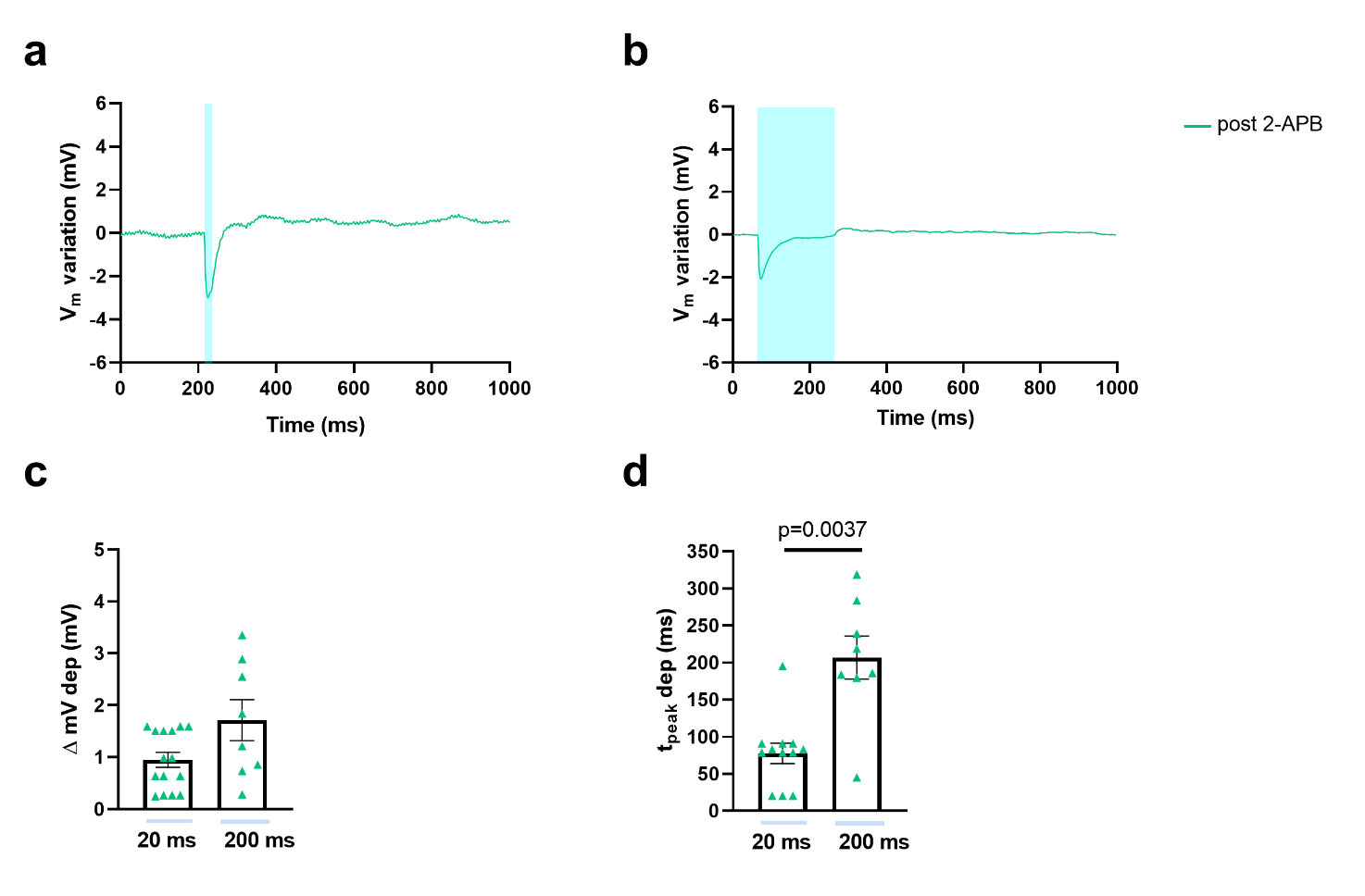
Figure S8: V_m_ depolarization is preserved in light-stimulated Ziapin2-loaded AMVMs upon treatment with 2-APB.*** *Representative whole-cell current-clamp traces recorded in cells loaded with Ziapin2 and stimulated with 20 ms- (panel a) or 200 ms-long (panel b) single light pulses, following 75 μM 2-APB treatment.* *Traces have been reported as relative V_m_ variation to better appreciate the light-induced effect; photoexcitation is represented by the cyan shaded area. Light power density was set at 50 mW/mm^2^. Peak depolarization (panel c, Δ_Vm_ dep 20 ms: 0.95 ± 1.14 mV, n = 15; Δ_Vm_ dep 200 ms: 1.71 ± 0.39 mV, n = 8) and time-to-peak of depolatization (panel d, t_peak_ dep 20 ms: 77.4 ± 13.6 ms, n = 8; t_peak_ dep 200 ms: 206.8 ± 29.1 ms, n = 8) in 25 μM Ziapin2-loaded AMVMs exposed to 75 μM 2-APB. Data are represented as mean ± SEM; N = 4. Statistical comparison was performed with the Mann-Whitney test.*

***
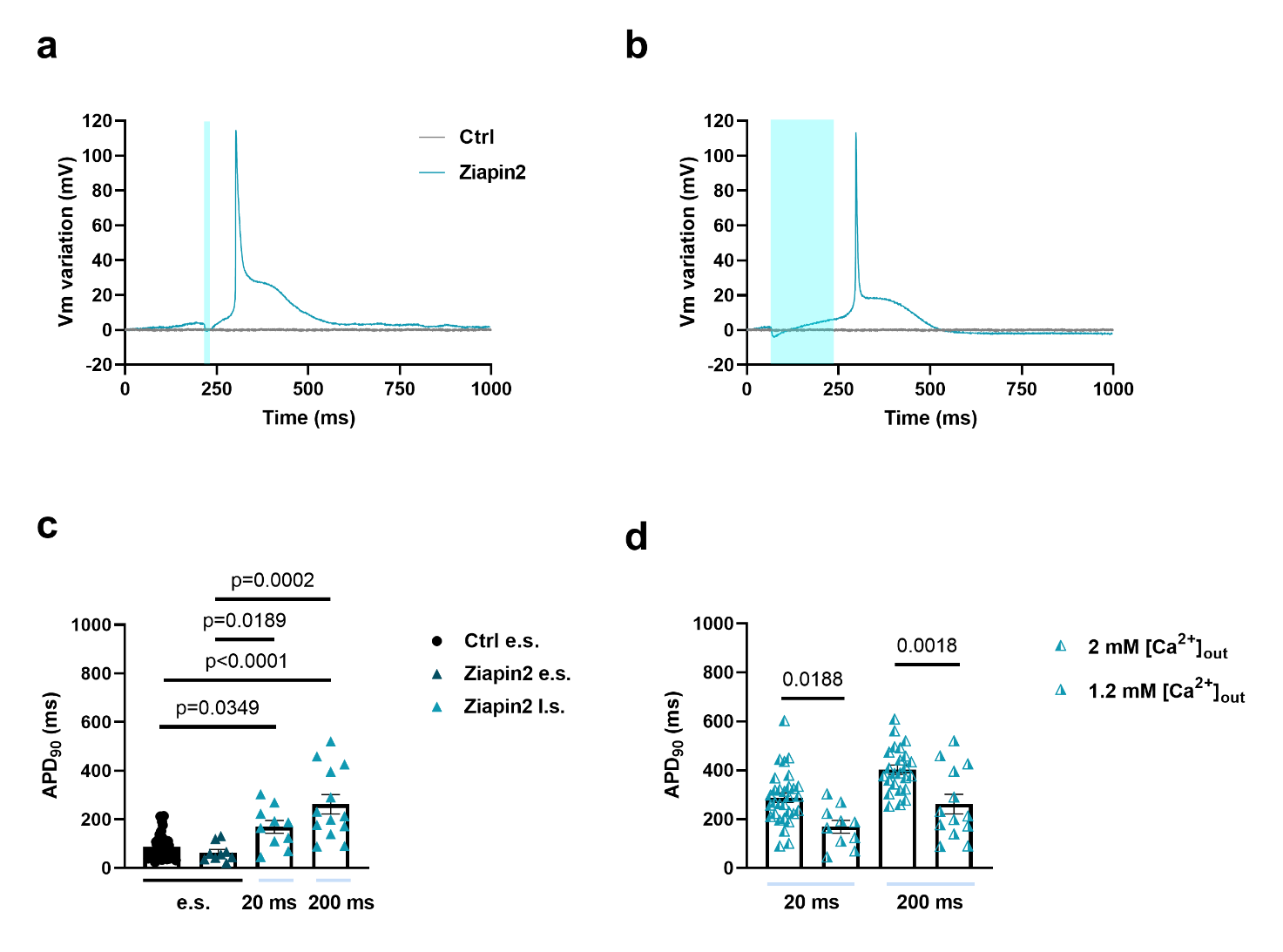
Figure S9: Photoinduced action potentials in Ziapin2-loaded AMVMs in presence of 1.2 mM extracellular Ca^2+^.*** *Representative action potentials (APs) recorded in AMVMs loaded with either vehicle (Ctrl, shown in gray) or 25 µM Ziapin2 (shown in teal) and stimulated with short (20 ms, panel a) or long (200 ms, panel b) single light pulses. Traces have been reported as relative V_m_ variation to better appreciate the light-induced effect; photoexcitation is represented by the cyan shaded area. Light power density was set at 50 mW/mm^2^. c) Comparison of action potential duration at 90% of repolarization (APD_90_) within electrically induced (APD_90_ Ctrl: 87 ± 8 ms, n = 39; APD_90_ Ziapin2: 63.9 ± 12.5 ms, n = 9) and light-evoked (APD_90_ 20 ms: 169.3 ± 26.4 ms, n = 10; APD_90_ 200 ms: 261.8 ± 39.9 ms, n = 13) action potentials. d) Comparison of APD_90_ within light-induced AP in presence of 2mM extracellular Ca^2+^ (APD_90_ 20 ms: 287.4 ± 19.6 ms, n = 30; APD_90_ 200 ms: 402 ± 19 ms, n = 24) or 1.2mM extracellular Ca^2+^ (APD_90_ 20 ms: 169.3 ± 26.4 ms, n = 10; APD_90_ 200 ms: 261.8 ± 39.9 ms, n = 13). Data are represented as mean ± SEM. Ctrl e.s. N = 13; Ctrl Ziapin2 e.s. N = 5;* *Ziapin2 20 ms N= 6; Ziapin2 200 ms N= 6 with 1.2 mM [Ca^2+^]_out_ and Ziapin2 20 ms N= 10; Ziapin2 200 ms N= 8 with 2 mM [Ca^2+^]_out_. Statistical comparisons were performed with the Kruskal-Wallis test (panel c) or the ordinary one-way ANOVA (panel d), depending on the normality of the data distribution.*
